# Stand-alone virtual reality exposure therapy as a treatment for social anxiety symptoms: a systematic review and meta-analysis

**DOI:** 10.48101/ujms.v128.9289

**Published:** 2023-09-14

**Authors:** Christian Rejbrand, Brynjar Fure, Karin Sonnby

**Affiliations:** a Faculty of Medicine and Health, School of Medical Sciences, Campus USÖ, Örebro, Sweden; b Centre for Clinical Research, Region Värmland, Karlstad, Sweden;; c Centre for Clinical Research, Region Västmanland, Västerås, Sweden

**Keywords:** Social fears, simulated reality, computer modelling, help

## Abstract

**Introduction:**

Social anxiety is common and can have far-reaching implications for affected individuals, both on social life and working performance. Usage of virtual reality exposure therapy (VRET) has gained traction. The aim of the present systematic review was to evaluate the effect of stand-alone VRET on social anxiety symptoms.

**Method:**

We searched systematically in *PubMed*, *Embase*, *PSYCinfo*, and *ERIC* in May 2022 for studies with participants with social anxiety symptoms receiving stand-alone VRET. Two reviewers independently selected relevant studies in a two-step procedure, and the risk of bias was assessed.

**Results:**

Of 158 hits, 7 studies were selected for full-text reading, 6 were chosen for evaluation, and 5 were included in meta-analyses. VRET resulted in a significantly lower anxiety score in treated individuals with a standard mean difference of −0.82, 95% confidence interval –1.52 to –0.13, compared to controls.

**Conclusion:**

Stand-alone VRET may reduce social anxiety symptoms. However, despite promising results, there is still uncertainty as the effect estimate is based on few studies with few participants each and a high risk of bias.

## Introduction

Symptoms of social anxiety are common, and the lifetime prevalence of social anxiety disorder (SAD) has been reported at 4% (1). The prevalence is particularly high in young women with low education and socioeconomic status in middle- and high-income countries (1–3).

Social anxiety disorder is categorised into *SAD* and *SAD, performance only* (DSM-5) (4). Individuals with the performance only subtype experience performance anxiety only when giving a public speech, taking a test, or attending a job interview (4).

Implications of SAD can be far-reaching, with affected individuals reporting higher rates of mood disorders, drug and alcohol abuse, and even suicide (4). Impairment in working life performance is also reported, leading to substantial societal costs as many refrain from seeking healthcare (5).

Treatment includes cognitive-behavioural therapy (CBT) and medication, with selective serotonin reuptake inhibitors (SSRIs) being first line (6). However, side effects such as impotence, nausea, and dry mouth limit their use (6). One limitation of CBT is poor compliance as much effort is required by the patient (6). To be physically exposed in environments with other people can be very stressful, even if it is done gradually. In such situations, it could feel reassuring to be able to abort with a push of a button. This is where virtual reality (VR) comes into the picture.

Virtual reality is a technology in which an environment is simulated, either using computer-generated imagery or real-life footage, such that the user experiences being in that environment visually and audially (7). This is done with the help of VR goggles and headphones (7). Having mainly been used in the gaming industry, technological advances have increased the fidelity of simulations, and its usage in exposure therapy (VRET) has been increasingly explored as an alternative to real-world exposure therapy (8–10). Several systematic reviews have been conducted; however, few have focused on SAD, and VRET has been administered as a component of CBT rather than stand-alone therapy, hence making it difficult to evaluate its effect (11).

The aim of the present systematic review and meta-analysis is therefore to evaluate stand-alone VRET as treatment for social anxiety symptoms. We hypothesised that stand-alone VRET will significantly reduce participants’ symptoms of social anxiety.

## Materials and methods

### Protocol and registration

This systematic review was conducted according to the Preferred Reporting Items for Systematic Reviews and Meta-Analyses (PRISMA) statement. The study was registered in PROSPERO, ID: CRD42022361900.

#### Type of studies

Only randomised controlled trials were eligible for inclusion due to their relatively high level of evidence. Studies had to be reported in either English or Swedish, with a study sample of more than 10 subjects. Two interventions were allowed: VRET and augmented reality exposure therapy (ARET). Augmented reality is a technology in which the user is in a real environment with computer-generated elements such as virtual avatars that are seen through goggles or a screen. It can be seen as a light version of VR and was therefore considered relevant to be included. Both VRET and ARET had to be stand-alone therapy for inclusion.

#### Types of participants and outcomes and measures

Participants had to meet the criteria for social phobia (DSM-IV) or social anxiety disorder (DSM-5) or score high on validated self-reports of either symptoms of social anxiety or public speaking anxiety, for example, the Brief Fear of Negative Evaluation Scale (FNE) (12) and Social Interaction Anxiety Scale (SIAS) (13). These instruments have high internal consistency and test-retest reliability (13, 14).

Outcomes were measured as changes between pre- and post-assessment of symptom levels in the intervention group compared to the comparison group. Eligible comparisons were waiting list, control, or CBT. This would enhance any potential effects of VRET and ARET, both in relation to no treatment and to CBT and hence in vivo exposure therapy.

### Information sources and literature search

Data sources used in the search included *PubMed*, *PSYCinfo*, *Embase*, and *ERIC*. An information specialist M.M. was consulted in constructing appropriate search terms and conducting the search. The data sources were chosen because *PubMed* is the largest database for medical studies and *PSYCinfo* is the largest regarding psychiatric diagnoses and mental health. *Embase* is also one of the major databases for medical studies. To supplement these sources, a final search was made in *ERIC*, which is a smaller database that contains many psychological studies.

### Study selection

All records from the searches were organised in Endnote and duplicates removed. The first author (C.R.) and the supervisor of this project (K.S.) then independently reviewed abstracts of all studies for relevance and read the relevant studies in full text to deem if they fulfilled the inclusion criteria. Disagreements on inclusion eligibility were resolved through discussion. See Appendix A for complete search strategy. No constraint on publication date was set for inclusion.

#### Data collection process

Extraction of study characteristics was made by authors C.R. and K.S. (see Table 1). Selection of studies and appropriate outcome measures for inclusion in meta-analysis were done by all three authors.

**Table 1:** Characteristics of included studies showing intervention type, control-comparison, type of VR technology, sample size at study start, gender and age distribution of the participants, outcome measures as well as results post-treatment and at follow-up in case there was a follow-up. Outcomes are reported as mean with standard deviation (SD) if not stated otherwise. Primary outcomes are in bold, if stated in the article.

Study	Intervention	Control	VR technology	Population size	Sex (% female)	Age interval	Outcome measures	Result post-treatment	Result follow-up
**Anderson et al. 2013 (** **15** **)**	VRET or EGT	WL	Not stated. Computer-generated footage.	97	62%	19–69 (*M*=39)	PRCS^a^ FNE-B^b^ BAT^i^ CGI-I^j^ Expectancy for treatment outcome scale WAI-SF^k^ Homework compliance CSQ-8^l^	FNE-B: I: 39.47 (10.70) C: 42.45 (10.07)	-
								Cohen’s *d*: FNE-B: 0.29	
**Anderson et al. 2017 (** **16** **)**	VRET	EGT	-	28	71%	19–69 (*M*=42)	PRCS^a^ FNE-B^b^ PGI^m^ BAT^i^		6-year: FNE-B: I: 35.77 (5.99) C: 34.36 (10.93)
**Kampmann et al. 2016 (** **17** **)**	VRET or in vivo exposure therapy	WL	Delft Remote Virtual Reality Exposure Therapy with Vizard v3.0 software package	60	63%	18–65 (*M*=37)	**LSAS-SR^d^** **FNE-B^b^** BAT^i^ DASS-21^n^ PDBQ^o^ EUROHIS-QOL 8-item index^p^	FNE-B: I: 36.05 (8.37) C: 36.44 (8.77)	3-month: FNE-B: 0.47
**Zainal et al. 2021 (** **18** **)**	VRET	WL	BehaVR with Pico Goblin headset. Real-life footage	44	77%	18–53 (*M*=23)	**SPDQ^e^** **SIAS^f^** **MASI^g^** PSWQ^q^ PHQ-9^r^ Qualitative feedback	SIAS: I: 25.29 (9.50) ITT C: 29.80 (13.28) ITT	Hedge’s *g*: SIAS: 3-month: -4.6 (p<.001) 6-month: -4.04 (p<.001) Post-3-month: -0.38 Post-6-month: -0.34
								Hedge’s *g*: SIAS: -4.58 (p<.001)	
**Lindner et al. 2019 (** **19** **)**	Therapist or self-guided VR	WL	Samsung gear VR headset on Galaxy Note 4/Google Cardboard with personal phone. Virtual speech app. Real-life footage.	50	72%	18+ (*M*=31)	**PSAS^h^** LSAS-SR^d^ FNE-B^b^ PHQ-9^r^ GAD-7^s^ BBQ^t^	PSAS: I: 56.29 (10.49) C: 69.28 (5.80)	-
								PSAS: Cohen’s *d*: 0.83	
**Reeves et al. 2021 (** **20** **)**	VRET with virtual audience or VRET without virtual audience	No treatment	Samsung Gear VR powered by Oculus with Samsung Galaxy S7. Real-life footage.	51	94%	18–45 (*M*=26)	PSAS^h^ LSAS-SR^d^ FNE-B^b^ IPQ^c^	-	10-week: I: 32.23 (6.73) audience C: 49.46 (9.34) audience

a Personal Report of Confidence as Speaker.

b Fear of Negative Evaluation – Brief Form.

c Igroup Presence Questionnaire.

d Liebowitz social anxiety scale – self-report.

e Social Phobia Diagnostic Questionnaire.

f Social Interaction Anxiety Scale.

g Measure of Anxiety in Selection Interviews.

h Public Speaking Anxiety Scale.

i Behavioral Avoidance Test.

j Clinicial Global Impressions of Improvement.

k Working Alliance Inventory – Short Form.

l Client Satisfaction Questionnaire.

m Patient Global Improvement.

n Depression Anxiety Stress Scale.

o Personality Disorder Belief Questionnaire.

p Eurohis Quality of Life Scale.

q Penn State Worry Questionnaire.

r Patient Health Questionnaire.

s Generalized Anxiety Disorder 7-item.

t Brunnsviken Brief Quality of Life Scale.

### Quality assessment and risk of bias

C.R. and K.S. independently assessed bias for each included article using the Critical Appraisal Skills Programme (CASP) (see Appendix B) (21). In case of disagreement, consensus was reached after discussion. In addition, the GRADE instrument was used to assess overall confidence in the pooled effect estimates across studies (22).

### Planned methods of analysis

The present systematic review aimed at evaluating included studies by narrative reporting of results and, when possible, meta-analyses. If three or more studies used a common outcome measure, these were pooled in a meta-analysis. Outcome measures were reported as mean difference (MD) with confidence intervals (CI) when studies used the same outcome measure and as standardised mean difference (SMD) with CI when different outcome measures were used across studies. Heterogeneity was calculated using *I*^2^. Due to high levels of heterogeneity of the included studies, the meta-analyses were performed with a random effects model. Meta-analyses were conducted using ReviewManager 5.4*.*

### Ethical considerations

As the study design was that of a systematic review and meta-analysis, no ethical approval was required. The present systematic review and meta-analysis was performed according to Swedish law and guidelines published by the Ethical Committee of Uppsala University, Sweden. We used only data from published studies available on the internet.

## Results

### Study selection

**Figure 1:**
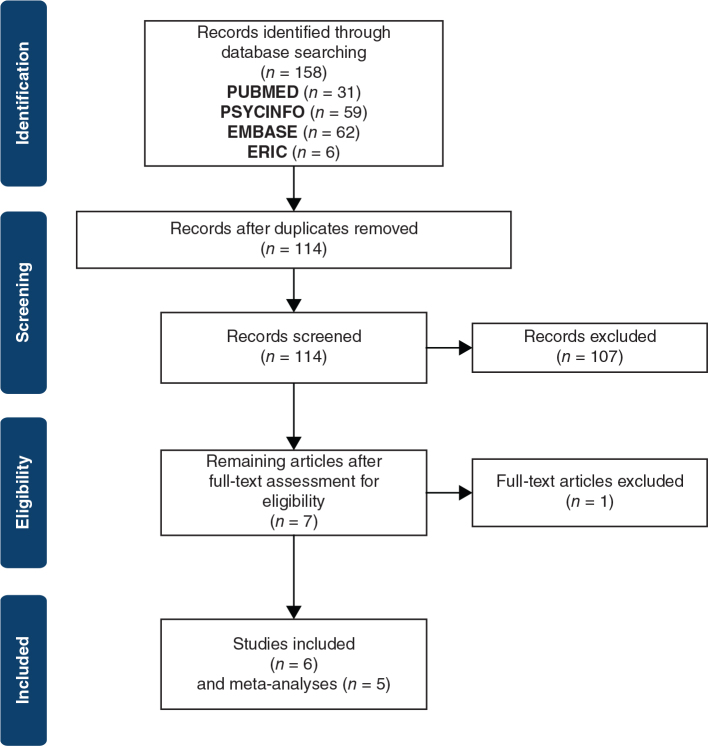
PRISMA flowchart of the search and review process.

The search in *PubMed* was conducted on June 15, 2022, and in *PSYCinfo*, *Embase,* and *ERIC* on June 16, 2022. In total, 158 records were identified, with 114 remaining after removing duplicates. After reviewing title and abstract, seven were included for full-text assessment. One study, Benbow (23) was excluded due to presumed double reporting of the sample included in the study by Anderson et al. (16), which was favoured for inclusion due to more participants. Of these six articles, five were selected for meta-analysis. The long-term follow-up study by Anderson et al. (16) used comparison with exposure group therapy (EGT) rather than no treatment, so results from this study were included only for narrative reporting. See Figure 1 for a flowchart of the selection process.

Participants in the studies were recruited through several digital and analogue means using newspaper and internet ads, flyers, referrals from professionals, and more. Common criteria for exclusion were concomitant medication with tranquillisers, non-stable antidepressant treatment, current alcohol or drug abuse, as well as a history of mania, psychosis, or schizophrenia.

### Study characteristics

There was a majority of female participants in all studies with a median age spanning from 23 to 42 years. Population size varied between 28 and 97. All studies were conducted in Western countries (the United States, the Netherlands, the United Kingdom, and Sweden). Study length ranged from 4 to 8 weeks, with eventual follow-up spanning from 10 weeks to 6 years. Study characteristics of included studies are presented in Table 1.

### Critical appraisal and risk of bias of included studies

Critical appraisal including risk of bias was assessed using a checklist for randomised controlled studies from the CASP (see Appendix B) (21). The checklist has four appraisal sections (A–D) and an appraisal summary. The assessment of each section is illustrated as a traffic light diagram, where green means low risk of bias, yellow medium risk of bias, and red high risk of bias (Table 2). All studies were deemed to have low risk of bias under section A due to randomisation and selection. The Anderson et al. (16) study could not be assessed as it was a follow-up to the study by Anderson et al. (15) parent study. All but one study (19) were considered to have high risk of bias in section B due to lack of pre-published study protocols. In section C, all studies were deemed to have medium risk of bias as most neither reported CI nor conducted cost-effectiveness analyses. Under section D, the generalizability of the results of all studies was judged to be comparable with other symptomatic mainly non-clinical populations. Overall, the appraisal summary for the applicability of the results in non-clinical populations with symptoms of social anxiety was considered uncertain if applicable, due to the total risk of bias in each study.

**Table 2:** Traffic light diagram illustrating the assessment of risk of bias of the included studies where green means low, yellow medium, and red high risk of bias. Black means ‘not applicable’.

Study	Section A: Is the basic study design valid for a randomised controlled trial?	Section B: Was the study methodologically sound?	Section C: Results: effects, precision, benefits?	Section D: Will the results help locally?	APPRAISAL SUMMARY: Conclusion about the paper? Would you use it to change your organisation?
Anderson et al. 2013	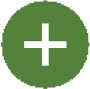	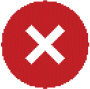	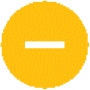	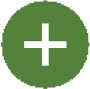	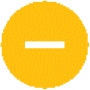
Anderson et al. 2017	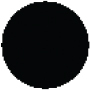	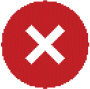	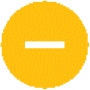	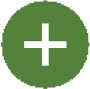	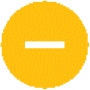
Kampmann et al. 2016	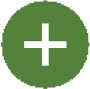	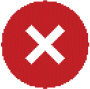	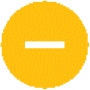	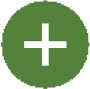	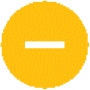
Zainal et al. 2021	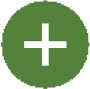	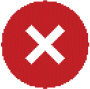	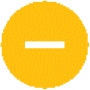	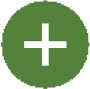	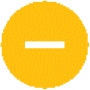
Lindner et al. 2019	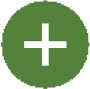	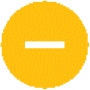	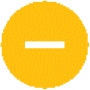	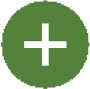	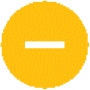
Reeves et al. 2021	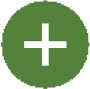	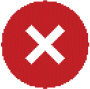	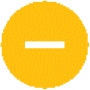	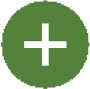	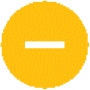

### Data synthesis

**Figure 2:**
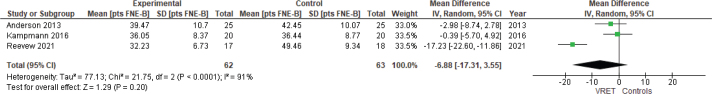
Forest plot showing mean difference of FNE-B points in individuals treated with VRET compared to controls. FNE-B: Fear of Negative Evaluation Scale brief version; SD: standard deviation; VRET: virtual reality exposure therapy.

The studies by Anderson et al. (15), Kampmann et al. (17), and Reeves et al. (20) all used FNE-B mean scores as outcome measure and were analysed together in a first meta-analysis. The results indicated a reduction of symptom severity (lower mean scores) for stand-alone VRET with MD –6.88 points (CI –17.31 to 3.55) compared to the control group; however, the result did not reach statistical significance and the heterogeneity was high (see Figure 2).

**Figure 3:**
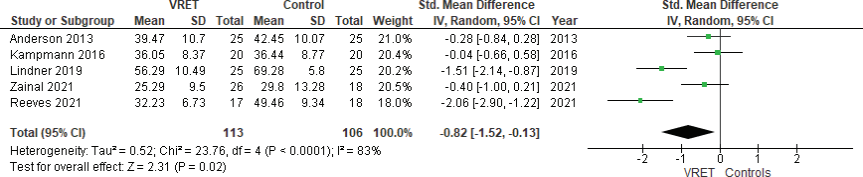
Forest plot showing standardised mean difference of social anxiety in individuals treated with VRET compared to controls. SD: standard deviation; VRET: virtual reality exposure therapy.

The second meta-analysis included five studies (15, 17–20) and three different outcome measures (FNE-B, PSAS, and SIAS) and yielded a significant reduction of symptoms of anxiety with SMD –0.82 (CI –1.52 to –0.13) in treated individuals compared to the control group, indicating a statistically significant reduction in symptom severity. Heterogeneity was somewhat lower than in the first analysis (see Figure 3).

After conducting the meta-analyses, it was noted in a corrigendum to the Lindner et al. (19) study that some of the scores reported were not correct due to a phrasing error and the author was contacted for a revised version (19). However, the updated numbers differed less than 1.1 points from the numbers used in the second meta-analysis, with the VRET group scoring lower at post-treatment as opposed to the control. This would only further enhance the already significant result of the study by Lindner et al. (19) in the second meta-analysis and not alter our conclusion. As such, no meta-analysis with the revised version was conducted.

### Narrative analysis

The study by Anderson et al. (16) compared long-term effects of VRET and EGT. The results showed a lasting reduction of symptoms of anxiety measured with FNE-B at follow-up in both groups, without significant difference between the two treatments.

### Confidence in the effect estimates from meta-analyses

The GRADE instrument (22) was used to assess our confidence in the pooled effect estimates across included studies (see Table 3). The three-study meta-analysis showed a low certainty of evidence meaning that further research is ‘very likely to have an important impact on the confidence in the estimate of effect and is likely to change the estimate’. The five-study meta-analysis showed a moderate certainty of evidence meaning that further research is ‘likely to have an important impact on the confidence of the estimate of effect and may change the estimate’.

**Table 3:** GRADE summary of findings table of meta-analyses.

	Certainty assessment							№ of patients		Effect		Certainty	Importance
Meta-analysis	No. of studies	Study design	Risk of bias	Inconsistency	Indirectness	Imprecision	Other considerations	VRET or ARET	No treatment or other treatment than VRET/ARET	Relative (95% CI)	Absolute (95% CI)		
I*	3	Randomised trials	Serious^a^	Not serious	Not serious	Serious^b^	None	62	63	-	MD **6.88 points lower** (17.31 lower to 3.55 higher)	⊕⊕◯◯ Low	IMPORTANT
II*	5	Randomised trials	Serious^a^	Not serious	Not serious	Serious^b^	Strong association	113	106	-	SMD **0.82 SD lower** (1.52 lower to 0.13 lower)	⊕⊕⊕◯ Moderate	IMPORTANT

Note. Question: VRET compared to no treatment or other treatment than VRET for symptoms of social anxiety.

a Risk of attrition bias. Protocol not pre-registered. Declaration of interests not reported.

b Few studies with few participants.

I* = Three-study meta-analysis on FNE-B. II* = Five-study meta-analysis on FNE-B, PSAS, and SIAS.

## Discussion

Altogether, the findings of this systematic review and meta-analysis of randomised controlled studies support the short- and long-term effectiveness of stand-alone VRET as treatment for symptoms of social anxiety. The literature on the area was sparse; however, all findings point in the same direction. The meta-analysis with the largest possible number of studies showed significant results, yet the one with only three studies included did not reach significance. Only one study evaluated and could show long-term effectiveness 4–6 years after treatment.

Several earlier meta-analyses have shown VRET to be effective when combined with CBT (24–26). Considering the results of the current meta-analysis, it is possible that stand-alone VRET is less effective than when combined with CBT. It is also possible that differences in study sample sizes are the reason for this discrepancy as earlier meta-analyses have included more studies and participants. Nevertheless, according to the Cochrane Handbook for Systematic Reviews, an SMD of 0.2 usually corresponds to a small effect, 0.5 to a moderate effect, and 0.8 to a large effect (27). The result of the present meta-analysis based on five studies, with an SMD of –0.82 in favour of the intervention group, should be considered a large effect. Although SMD cannot be directly translated into a clinically relevant effect, a large SMD corresponds to a low number needed to treat and a high probability of benefit, and thus, it is likely to be associated with a clinical effect (28). Finally, the narrative analysis of Anderson et al. (16) showed similar ability of VRET and EGT in reducing social anxiety symptoms at long-term follow-up 4–6 years after initial treatment.

The results of the present systematic review must be interpreted in the light of several limitations. Firstly, the number of studies and participants included was low, making the result of the meta-analyses less certain and increases influence of random findings. Secondly, risk of bias of attrition and reporting was high in most of the included studies due to high dropout rates and a lack of pre-publicised study protocols. According to GRADE, our confidence in the effect estimates is low and moderate indicating that further research is likely to alter the results.

A strength of the present systematic review was the methodology following the PRISMA statement, which enhances comparison with former and future similar studies. One further strength was the enrollment of only randomised controlled studies, which decreases some risks of bias, e.g. due to selection of participants. Moreover, the participants were mainly young females from Europe and North America, which are populations with high prevalence of symptoms of social anxiety. This may enhance generalizability of the results to these symptomatic, mainly non-clinical populations.

## Conclusion

Stand-alone VRET showed ability to significantly reduce symptoms of social anxiety both on short and long terms. Based on the present systematic review with a sparse number of studies, the results are uncertain, however, still not advising against further research. Yet, much is unknown as for example the influence of different types of VR technology, type of treatment programmes, and if other populations, e.g. patients from primary care or psychiatric settings, would benefit from treatment with VRET.

## Appendix A: Search results

**Table A.1:** Results from search on PubMed.

PubMed 2022-06-15 search	Search terms	Filter	Number of hits
**1**	‘Phobia, Social’ [MeSH]		1,077
**2**	‘social anxiety’ [title/abstract]		7,273
**3**	Sad [title/abstract]		11,286
**4**	‘social phobia’ [title/abstract]		4,177
**5**	‘public speaking anxiety’ [title/abstract]		81
**6**	‘Virtual Reality Exposure Therapy’ [MeSH]		824
**7**	‘Virtual Reality’ [MeSH]		4,521
**8**	‘virtual Reality’ [title/abstract]		14,223
**9**	‘VRET’ [title/abstract]		120
**10**	‘VR’ [title/abstract]		10,762
**11**	‘Augmented Reality’ [MeSH]		829
**12**	‘augmented reality’ [title/abstract]		3,393
**13**	‘AR’ [title/abstract]		59,566
**14**	#1 OR #2 OR #3 OR #4 OR #5		20,090
**15**	#6 OR #7 OR #8 OR #9 OR #10 OR #11 OR #12 OR #13		23,406
**16**	#14 AND #15		147
**17**	#16 AND (‘randomized controlled trial’ OR rct)		31
**18**	#16 limit: randomized controlled trial	Randomized Controlled Trial	24
**19**	#17 OR #18		31

**Table A.2:** Results from search on PSYCinfo.

PSYCinfo 2022-06-25 search	Search terms	Number of hits
**1**	exp Social Anxiety/	5,592
**2**	exp Social Phobia/	4,999
**3**	speech anxiety/	611
**4**	social anxiety disorder.ab,ot,ti.	3,541
**5**	social phobia.ab,ot,ti.	5,314
**6**	public speaking anxiety.ab,ot,ti.	259
**7**	#1 or #2 or #3 or #4 or #5 or #6	13,082
**8**	exp Virtual Reality Exposure Therapy/	229
**9**	exp Augmented Reality/	776
**10**	virtual reality exposure therapy.ab,ot,ti.	258
**11**	augmented reality.ab,ot,ti.	1,121
**12**	#8 OR #9 OR #10 OR #11	1,492
**13**	#7 AND #12	57
**14**	exp randomized controlled trials/ or rct.ab,ot,ti. or randomized controlled trial.ab,ot,ti.	25,650
**15**	#13 AND #14	10

**Table A.3:** Results from search on Embase.

Embase 2022-06-26 search	Search terms	Number of hits
**1**	’social phobia’/exp OR ’social phobia’	15,084
**2**	’social anxiety’/exp OR ’social anxiety’	9,586
**3**	’public speaking anxiety’	104
**4**	’speech anxiety’	96
**5**	’virtual reality exposure therapy’/exp OR ’virtual reality exposure therapy’	957
**6**	’virtual reality’/exp OR ’virtual reality’	29,606
**7**	’vret’	154
**8**	’augmented reality’/exp OR ’augmented reality’	4,284
**9**	#1 OR #2 OR #3 OR #4	19,056
**10**	#5 OR #6 OR #7	29,612
**11**	#9 AND #10	272
**12**	#9 AND #10 AND [randomized controlled trial]/lim	40
**13**	#9 AND #10 AND (’randomized controlled trial’ OR rct)	62
**14**	#12 OR #13	62

**Table A.4:** Results from search on ERIC.

ERIC 2022-06-16 search	Search terms	Number of hits
**1**	TI social anxiety OR AB social anxiety	1,444
**2**	TI social phobia OR AB social phobia	234
**3**	TI public speaking anxiety OR AB public speaking anxiety	146
**4**	TI virtual reality OR AB virtual reality	1,412
**5**	TI virtual reality exposure therapy OR AB virtual reality exposure therapy	3
**6**	TI VRET OR AB VRET	2
**7**	TI augmented reality OR AB augmented reality	718
**8**	#1 OR #2 OR #3	1,662
**9**	#4 OR #5 OR #6 OR #7	1,995
**10**	#8 AND #9	6

## Appendix B: CASP

**CASP Randomised Controlled Trial Standard Checklist:**

11 questions to help you make sense of a randomised controlled trial (RCT)

**Main issues for consideration:** Several aspects need to be considered when appraising a randomised controlled trial:

Is the basic study design valid for a randomised controlled trial? (Section A) Was the study methodologically sound? (Section B) What are the results? (Section C) Will the results help locally? (Section D)

The 11 questions in the checklist are designed to help you think about these aspects systematically.

**How to use this appraisal tool:** The first three questions (Section A) are screening questions about the validity of the basic study design and can be answered quickly. If, in light of your responses to Section A, you think the study design is valid, continue to Section B to assess whether the study was methodologically sound and if it is worth continuing with the appraisal by answering the remaining questions in Sections C and D.

Record ‘Yes’, ‘No’ or ‘Can’t tell’ in response to the questions. Prompts below all but one of the questions highlight the issues it is important to consider. Record the reasons for your answers in the space provided. As CASP checklists were designed to be used as educational/teaching tools in a workshop setting, we do not recommend using a scoring system.

**About CASP Checklists:** The CASP RCT checklist was originally based on JAMA Users’ guides to the medical literature of 1994 (adapted from Guyatt GH, Sackett DL and Cook DJ) and piloted with healthcare practitioners. This version has been updated taking into account the CONSORT 2010 guideline (http://www.consort-statement.org/consort-2010, accessed 16 September 2020).

**Citation:** CASP recommends using the Harvard style, i.e. Critical Appraisal Skills Programme (2021). CASP (insert name of checklist, i.e. Randomised Controlled Trial) Checklist. [online] Available at: insert URL. Accessed: insert date accessed.

©CASP this work is licensed under the Creative Commons Attribution – Non-Commercial- Share A like. To view a copy of this licence, visit https://creativecommons.org/licenses/by-sa/4.0/

Critical Appraisal Skills Programme (CASP) www.casp-uk.net Part of OAP Ltd

**Study and citation: ………………………………………………………………………………………………………………………………**

**Table:**

**Section A:** Is the basic study design valid for a randomised controlled trial?				
**1**.	**Did the study address a clearly focused research question?** *CONSIDER:* *Was the study designed to assess the outcomes of an intervention?* *Is the research question ‘focused’ in terms of:* *Population studied* *Intervention given* *Comparator chosen* *Outcomes measured?*	Yes □	No □	Can’t tell □
**2**.	**Was the assignment of participants to interventions randomised?** *CONSIDER:* *How was randomisation carried out? Was the method appropriate?* *Was randomisation sufficient to eliminate systematic bias?* *Was the allocation sequence concealed from investigators and participants?*	Yes □	No □	Can’t tell □
**3**.	**Were all participants who entered the study accounted for at its conclusion?** *CONSIDER:* *Were losses to follow-up and exclusions after randomisation accounted for?* *Were participants analysed in the study groups to which they were randomised (intention-to-treat analysis)?* *Was the study stopped early? If so, what was the reason?*	Yes □	No □	Can’t tell □
**Section B:** Was the study methodologically sound?				
**4**.	**Were the participants ‘blind’ to intervention they were given?** **Were the investigators ‘blind’ to the intervention they were giving to participants?** **Were the people assessing/analysing outcome/s ‘blinded’?**	Yes □ □ □	No □ □ □	Can’t tell □ □ □
**5**.	**Were the study groups similar at the start of the randomised controlled trial?** *CONSIDER:* *Were the baseline characteristics of each study group (e.g. age, sex, socioeconomic group) clearly set out?* *Were there any differences between the study groups that could affect the outcome/s?*	Yes □	No □	Can’t tell □
**6**.	**Apart from the experimental intervention, did each study group receive the same level of care (that is, were they treated equally)?** *CONSIDER:* *Was there a clearly defined study protocol?* *If any additional interventions were given (e.g. tests or treatments), were they similar between the study groups?* *Were the follow-up intervals the same for each study group?*	Yes □	No □	Can’t tell □
**Section C:** What are the results?				
7.	**Were the effects of intervention reported comprehensively?** *CONSIDER:* *Was a power calculation undertaken?* *What outcomes were measured, and were they clearly specified?* *How were the results expressed? For binary outcomes, were relative and absolute effects reported?* *Were the results reported for each outcome in each study group at each follow-up interval?* *Was there any missing or incomplete data?* *Was there differential dropout between the study groups that could affect the results?* *Were potential sources of bias identified?* *Which statistical tests were used?* *Were p values reported?*	Yes □	No □	Can’t tell □
8.	**Was the precision of the estimate of the intervention or treatment effect reported?** *CONSIDER:* *Were confidence intervals (CIs) reported?*	Yes □	No □	Can’t tell □
9.	**Do the benefits of the experimental intervention outweigh the harms and costs?** *CONSIDER:* *What was the size of the intervention or treatment effect?* *Were harms or unintended effects reported for each study group?* *Was a cost-effectiveness analysis undertaken? (Cost-effectiveness analysis allows a comparison to be made between different interventions used in the care of the same condition or problem.)*	Yes □	No □	Can’t tell □
**Section D:** Will the results help locally?				
**10**.	**Can the results be applied to your local population/in your context?** *CONSIDER:* *Are the study participants similar to the people in your care?* *Would any differences between your population and the study participants alter the outcomes reported in the study?* *Are the outcomes important to your population?* *Are there any outcomes you would have wanted information on that have not been studied or reported?* *Are there any limitations of the study that would affect your decision?*	Yes □	No □	Can’t tell □
**11.**	**Would the experimental intervention provide greater value to the people in your care than any of the existing interventions?** *CONSIDER:* *What resources are needed to introduce this intervention taking into account time, finances, and skills development or training needs?* *Are you able to disinvest resources in one or more existing interventions in order to be able to re-invest in the new intervention?*	Yes □	No □	Can’t tell □

**APPRAISAL SUMMARY:** *Record key points from your critical appraisal in this box. What is your conclusion about the paper? Would you use it to change your practice or to recommend changes to care/interventions used by your organisation? Could you judiciously implement this intervention without delay?*
